# Timing of Early Postoperative MRI following Primary Glioblastoma Surgery—A Retrospective Study of Contrast Enhancements in 311 Patients

**DOI:** 10.3390/diagnostics13040795

**Published:** 2023-02-20

**Authors:** Alexander Malcolm Rykkje, Vibeke Andrée Larsen, Jane Skjøth-Rasmussen, Michael Bachmann Nielsen, Jonathan Frederik Carlsen, Adam Espe Hansen

**Affiliations:** 1 Department of Diagnostic Radiology, Copenhagen University Hospital, Rigshospitalet, 2100 Copenhagen, Denmark; 2 Department of Clinical Medicine, University of Copenhagen, 2200 Copenhagen, Denmark; 3 Department of Neurosurgery, Copenhagen University Hospital, Rigshospitalet, 2100 Copenhagen, Denmark

**Keywords:** Glioblastoma, magnetic-resonance imaging, early postoperative MRI, postoperative period, postoperative enhancement, time window, neuroimaging

## Abstract

An early postoperative MRI is recommended following Glioblastoma surgery. This retrospective, observational study aimed to investigate the timing of an early postoperative MRI among 311 patients. The patterns of the contrast enhancement (thin linear, thick linear, nodular, and diffuse) and time from surgery to the early postoperative MRI were recorded. The primary endpoint was the frequencies of the different contrast enhancements within and beyond the 48-h from surgery. The time dependence of the resection status and the clinical parameters were analysed as well. The frequency of the thin linear contrast enhancements significantly increased from 99/183 (50.8%) within 48-h post-surgery to 56/81 (69.1%) beyond 48-h post-surgery. Similarly, MRI scans with no contrast enhancements significantly declined from 41/183 (22.4%) within 48-h post-surgery to 7/81 (8.6%) beyond 48-h post-surgery. No significant differences were found for the other types of contrast enhancements and the results were robust in relation to the choice of categorisation of the postoperative periods. Both the resection status and the clinical parameters were not statistically different in patients with an MRI performed before and after 48 h. The findings suggest that surgically induced contrast enhancements are less frequent when an early postoperative MRI is performed earlier than 48-h, supporting the recommendation of a 48-h window for an early postoperative MRI.

## 1. Introduction

Following surgery for Glioblastoma, an early postoperative magnetic resonance imaging (MRI) is usually performed to evaluate the extent of the surgical resection [1,2]. The radiological assessment of the contrast-enhancing residual tumour on an early postoperative MRI is essential to provide a baseline for future assessments of the treatment responses to guide therapeutic decision-making [3]. Following the criteria by the Response Assessment in Neuro-Oncology (RANO) working group, enhancing residual tumour on an early postoperative MRI is categorised as either measurable or non-measurable [4]. In addition, because patient survival seems correlated with the extent of the tumour resection [5,6,7], the early postoperative assessment of a residual tumour could be important for the patient prognosis.

Surgically induced contrast enhancements can appear as a direct consequence of surgery itself either immediately or in the days and weeks following surgery [8,9,10]. Early thin linear contrast enhancements immediately bordering the resection cavity are often interpreted as surgically induced [11]. Such reactive enhancements typically increase in volume and intensity and may persist for weeks; thus, rendering the radiological assessment difficult [3,10]. Because of this, it has long been recommended that an early postoperative MRI is to be acquired within 72-h after surgery. However, surgically induced contrast enhancements can also appear within the 72-h window [11,12,13]. Recent guidelines by the European Association of Neuro-Oncology (EANO) suggest a 24–48-h window [14,15] based on a single study [12]. Hence, an update on the current practice would benefit from further evidence on the timing of an early postoperative MRI.

In this retrospective, observational study, we offer an analysis of different patterns of contrast enhancements during the early postoperative MRI following Glioblastoma surgery in a large cohort of patients. The aim is to investigate the timing of the early postoperative MRI within the 72-h window. We hypothesise that contrast enhancements induced by surgery will occur increasingly frequent for each postoperative day. 

## 2. Materials and Methods

### 2.1. Patient Population

Patients undergoing primary surgery for histology-verified Glioblastoma (following the 2016 WHO classification of CNS tumours) between November 2016 and March 2020 were retrospectively and consecutively included. Surgeries were identified using the neurosurgical planner on a day-to-day basis. The early postoperative MRI of patients was analysed. Access to preoperative and early postoperative MRIs before and after the contrast injection were necessary for inclusion and the early postoperative MRIs had to include a description of the resection status. Approval for the study was granted by the National Ethics Committee (protocol code 2101886).

### 2.2. MRI

Postoperative MRIs were performed on either 1.5 or 3 Tesla scanners. The MRI series acquired before the contrast injection were 2D sagittal T1-weighted (spin echo, slice thickness: 5 mm), 2D coronal T2-FLAIR (slice thickness 4 or 5 mm) and axial diffusion weighted (b-values of 0 and 1000, slice thickness 4 or 5 mm) with computation of the apparent diffusion coefficient. After the contrast injection (Gadovist, Bayer AB) the sequences were 2D axial T2 (radial sampling with Blade/Propeller, slice thickness 5 mm) and 3D sagittal T1 (gradient echo, slice thickness: 1 mm). 

### 2.3. Timing of Early Postoperative MRI

For each patient, the hours, and minutes from surgery (end of procedure) to the early postoperative MRI (first MR sequence) were extracted from the records for analysis. The time from surgery until the early postoperative MRI was then categorised into periods of time. Earlier studies have categorised timing in different ways, according to either imaging on the postoperative days 1, 2, and 3 (0–24, 25–47, and 48-h) [16,17], within 24-h [18,19], or before and after 45-h [12]. The recent EANO guidelines recommend acquiring an early postoperative MRI before 48-h after surgery; hence, dividing according to this time point was also of interest and was chosen as our primary endpoint. To ensure the robustness of our findings, other periods of time (0–24, 25–47, and 48-h; before and after 45-h; and 0–36, 37–60, and 60-h) were included for statistical analysis. As for the latter, when observing the timing of the early postoperative MRI, we chose to have three periods of time dividing at 36- and 60-h from surgery (see Results section below). 

### 2.4. Image Analysis

**Figure 1:**
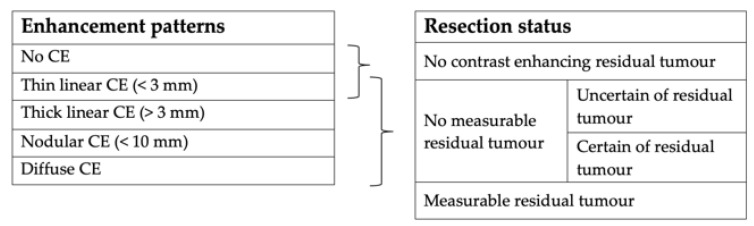
Overview of the data categorisation process. Enhancement patterns are specified in the left table with corresponding resection status specified in the table to the right. Several types of contrast enhancements could be present in one patient. Abbreviations: contrast enhancement (CE).

The data were categorised in two ways, one describing the enhancement patterns and one describing the resection status (Figure 1). 

**Figure 2:**
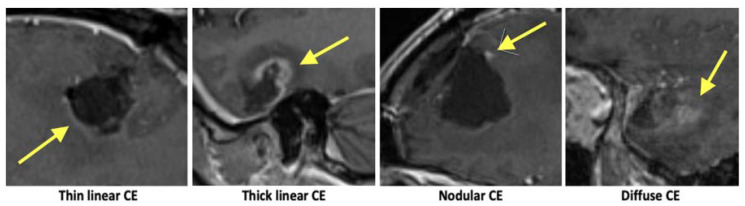
Examples of contrast enhancement. Enhancement patterns are marked with yellow arrows.

Inspired by earlier practices, the contrast enhancements surrounding the resection cavity were examined and categorised based on their pattern as follows: no contrast enhancement, thin linear (<3 mm), thick linear (>3 mm), nodular (<10 mm), or diffuse (Figure 2) [12,18]. The resection status was divided into 3 groups, following recommendations by the RANO working group: no contrast enhancing residual tumour, non-measurable residual tumour, or measurable tumour [20]. Since it is not always clear if the contrast enhancements in the ‘non-measurable residual tumour’ group are in fact caused by the residual tumour, this group was subdivided into those a neuroradiologist would classify as either certain or uncertain of a residual tumour. As all patients in the ‘measurable tumour’ group had a residual tumour, they were excluded for the analysis of the enhancement patterns. For the remaining patients, the enhancement patterns were noted as present or not present with several patterns possible for each patient.

All definitions were agreed to jointly after reviewing several cases. A radiologist with 3-years of experience (A.M.R.) then received training in evaluating the early postoperative MRI before evaluating all the MRI scans. All cases were evaluated under the guidance of two board-certified neuroradiologists with 12- (J.F.C.) and 25- (V.A.L.) years of experience and were blinded to the time from surgery to the early postoperative MRI. 

### 2.5. Recorded Patient Clinical Parameters

The participants age, sex, and biopsy results were recorded. Furthermore, the clinical parameters, including the early warning score (EWS) and the Glasgow coma scale (GCS) until 48-h post-surgery were logged to assess if these influenced the time until the early postoperative MRI. The EWS is based on the vital signs of the patient with an aggregate score of 0–4 being considered low, 5–6 medium, and 7-high risk of clinical deterioration [21]. The GCS scores a patient’s level of consciousness, with scores of 3–8 being severe, 9–12 moderate, and 13–15 mild or no brain injury [22]. For our study, scores were grouped according to the abovementioned clinical practices. 

### 2.6. Statistical Analysis

The software used for the statistical analyses was IBM SPSS Statistics version 28.0. The Chi-squared (X^2^) test was performed to compare the findings at the different time intervals. The *p*-value was defined as significant if <0.05.

## 3. Results

The early postoperative MRIs of 311 patients were examined (125 female and 186 male). The mean age was 63.1 years ranging from 20 to 87. The early postoperative MRIs were performed at 1.5 T for 281 patients (90.4%) or 3 T for 30 patients (9.6%). These proportions between the field strengths did not change with time from surgery to the early postoperative MRI.

### 3.1. Timing

**Figure 3:**
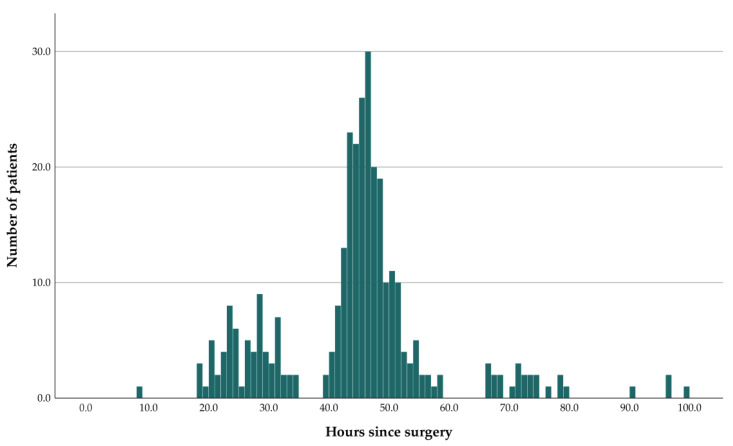
Histogram showing the time distribution of the early postoperative MRI for all patients. The x-axis represents the time from surgery to early postoperative MRI and the y-axis represents the number of patients for each hour.

The mean time from surgery to the early postoperative MRI was 44.5-h ranging from 8.3- to 99.7-h (Figure 3). As presented in Figure 3, most patients were examined with the postoperative MRI between 36- and 60-h after surgery (n = 217). This corresponds with postoperative day 2, and while some are performed before or after that time depending on when the scanner was available, most of the MRIs were acquired around day 2 at our institution. The gaps in the histogram reflect that both the surgery and the MRIs are not usually performed at night. Considering the distribution of the time from surgery to the postoperative MRI, a natural choice of categorisation of the timing is 0–36, 36–60 and 60–h, which is included in the analysis below.

### 3.2. Contrast Enhancements

Excluding the 47 patients with a measurable tumour contrast enhancement, a total of 264 MRI examinations were analysed (Table 1). The MRIs with no contrast enhancements significantly declined from 41/183 (22.4%) within 48-h post-surgery to 7/81 (8.6%) beyond 48-h post-surgery. The frequency of the thin linear contrast enhancements significantly increased from 99/183 (50.8%) within 48-h to 56/81 (69.1%) beyond 48-h. No significant differences were found for other types of contrast enhancements.

When categorising patients using three periods of time (according to the MRIs performed 0–36, 37–59, and 60-h after surgery), the MRIs with no contrast enhancements decreased significantly from 22/60 (36.7%) in period 1 (0–36-h) to 1/22 (4.5%) in period 3 (60-h). The presence of thin linear contrast enhancements increased from 24/60 (40%) in period 1 to 17/22 (77.3%) in period 3. Diffuse contrast enhancements increased significantly from 7/60 (11.7%) in period 1 and 34/182 (18.7%) in period 2 to 10/22 (45.5%) in period 3. The remaining types of contrast enhancements were not significantly different. 

If categorising a postoperative MRI according to the time intervals of 0–24, 25–47, and 48-h or before and after 45-h, similar results to the categorisation of before and after 48-h were found. A time-dependent decrease in MRIs with no contrast enhancements and an increase in thin linear enhancements were significant for both categorisations of the postoperative periods whilst other types of contrast enhancement were not. 

### 3.3. Resection Status

A total of 90 patients were described with no contrast enhancing tumours by the radiologist (Table 2). 

Furthermore, 174 had no measurable enhancing tumour and 47 had measurable enhancing tumour. Although not significant according to the primary endpoint (before and after 48-h), the frequency of patients with no contrast enhancing tumours decreased with time for all choices of categorisations of the postoperative periods. Only when dividing at 36- and 60-h were these differences significant, from 28/69 (40.6%) at 0–36-h, 58/217 (26.7%) at 37- to 59-h to 4/25 (16%) at 60-h, *p* = 0.029. No significant changes were seen for any other groups (no measurable tumour and measurable tumour) regardless of how the postoperative periods were defined. For the ‘no measurable’ group the radiologists were less frequently certain for any residual tumour for the MRI performed after 48-h; however, these differences were not significant. 

### 3.4. Clinical Parameters

The GCS and the EWS were logged to assess their influence on the time to the postoperative MRI. Consciousness was minimally affected or normal (GCS 13–15) for 289/311 (92.9%) of patients. No significant differences were found when comparing the GCS for patients with a postoperative MRI within 48-h to beyond 48-h. Significant differences were only found when dividing at 36- and 60-h; whereby, the frequencies of patients with minimal brain injury decreased with time, *p* = 0.001. For the EWS the highest aggregate score was 5 while 310/311 (99.7%) scored 0–4 (low risk of clinical deterioration) and for this reason no further statistical analysis was performed for this parameter. 

## 4. Discussion

This study demonstrates a significantly increased occurrence of thin linear contrast enhancements with time in the 72-h following primary Glioblastoma surgery. Correspondingly, the MRI scans without any contrast enhancements significantly decreased with time. The findings are consistent with the temporal development of surgically induced reactive contrast enhancements and support the recommendation of a 48-h window for an early postoperative MRI.

The recent EANO guidelines recommend acquiring a postoperative MRI within 48-h post-surgery [15], shortening the earlier recommended 72-h window. Accordingly, in our study, we chose differences in the occurrence of contrast enhancements before and after 48-h post-surgery as the primary endpoint. The update in the EANO guidelines relies on a single study by Bette et al. [12] with 173 patients; hence, our study of a larger number of patients adds to the current evidence for the shortening of the postoperative window for an MRI. The percentage point increase in examinations in our study showing thin linear contrast enhancements (from 50.8% within 48-h to 69.1% beyond-48-h) is comparable to that of Bette et al. (from 24.1% before 45-h to 45.5% after 45-h). However, we found the overall number of thin linear enhancements to be higher, which may reflect different radiological conventions for positivity of enhancements.

Earlier studies have employed a variety of categorisations of postoperative time periods within the 72-h postoperative window [12,16,18]. This study chose to divide at 48-h post-surgery as the primary endpoint but included three other periods of time for analysis to verify the robustness of the results and the results were robust to the choice of categorisation of the postoperative periods. An exception was for diffuse contrast enhancements, which increased significantly only when dividing at 36- and 60-h. However, the increase was caused by a relatively small number of patients with diffuse enhancement after 60-h.

Our study further explores the resection status of patients as defined by the RANO working group. Contrary to the analysis of contrast enhancements the resection status did not seem to be influenced by the timing of the early postoperative MRI for any categorisation of the postoperative time periods. Again, the exception is when dividing at 36- and 60-h where the ‘no contrast enhancing tumour’ group significantly decreased with time, *p* = 0.029. The explanation could be the increasing number of contrast enhancements with time, further highlighting the benefits of performing an MRI earlier than 48-h. The clinical parameters were recorded to investigate if an MRI acquired late were because of a clinical deterioration. Again, significant differences were only seen when dividing at 36- and 60-h, with a markedly lower GCS for patients scanned after 60-h. This suggests that the scan is sometimes performed later when the consciousness of the patient is affected. 

In this study, several categorisations of the postoperative periods in time were examined with similar results reported for all the periods of time. The novel categorisation with three periods dividing at 36- and 60-h were included because neither surgery nor the postoperative MRI is usually performed immediately after surgery or at night. This can be seen in the histogram (Figure 3) as a few hours around the 36- and 60-h mark with no postoperative MRI. We argue that this is a more practical divide than the 24- or 48-h divide often mentioned in the literature, since these would occur during the working day. Still, our findings are in line with the current guidelines recommending an early postoperative MRI within 24–48-h of surgery [15].

Our study suffered some limitations. Firstly, distinguishing between a residual tumour and a surgically induced reactive enhancement can be challenging and has not been pursued in this article. Bette et al. performed this by comparing early postoperative MRIs with a follow-up MRI after an initiation of oncological treatment and classified 66.1% of linear enhancements as reactive [12]. It is challenging to characterise the development of contrast enhancements over time and during radio- and chemotherapy, and this was not pursued in the current study. Secondly, as documented by earlier studies, reactive changes are likely to increase in intensity in the early days and weeks beyond the 72-h window, yet the current study made no attempt to follow these later developments of the contrast enhancements [3,23]. While beyond the scope of the current study, in a future study we aim to compare the early postoperative MRIs with the MRIs acquired before radiotherapy a few weeks later. Finally, as an observational study we had no influence over the timing of the early postoperative MRIs and the majority (n = 217) were performed between 36- and 60-h after surgery. Only a limited number of cases (n = 25) were available after 60-h, yet several significant changes occurred only in this postoperative period. For this reason and because the clinical parameters seemed to differ after 60-h, these radiological findings should be interpreted with care.

This study argues that early postoperative MRIs acquired before 48-h is preferable based on the occurrence of contrast enhancements. However, some studies suggest that the scan could be as early as during or immediately following surgery [24,25,26]. However, contrast-agent leaking into the resection cavity has been reported for intraoperative MRIs, which generally seemed to decrease or resolve on early postoperative MRIs [8]. Future studies should investigate whether intraoperative MRIs or early postoperative MRIs are preferable for assessing the extent of the resection. Finally, although survival amongst patients with Glioblastoma is thoroughly researched in the literature, it would be interesting for a future study to determine whether different types of contrast enhancements can add prognostic information to the already established clinical and molecular prognostic factors [27,28]. Additionally, while the extent of the resection and the effect on survival remains a controversial question [7,15,29,30], it has not previously been studied in relation to the resection status as defined by the RANO working group.

## 5. Conclusions

In the context of Glioblastoma surgery, our study found a significantly larger proportion of early postoperative MRI scans with thin linear enhancements when the MRI was performed more than 48-h post-surgery when compared to within 48-h post-surgery. Likewise, the proportion of MRI examinations with no contrast enhancements was smaller beyond 48-h post-surgery. Similar results were found for other divisions of the 72-h postoperative window. The results suggest that surgically induced contrast enhancements are less frequent the earlier the postoperative MRI is acquired. The study adds further evidence to support the recommendation of a 48-h window for early postoperative MRIs.

## Figures and Tables

**Table 1:** Top: Distribution of different contrast enhancements before and after 48-h. Bottom: Distribution of different contrast enhancements for three periods of time (0–36, 37–59, 60-h). More than one type of contrast enhancement can be present in one patient. Patients with measurable tumour were excluded from analysis.

CE (48-h DIVIDE)	0–47 h				48-h		*p*-Value (Chi-Sq)
	Patients, n (of Total)		Present (%)		Patients, n (of Total)	Present (%)	
No CE	41/183		22.4%		7/81	8.6%	0.008
Thin linear	93/183		50.8%		56/81	69.1%	0.006
Thick linear	66/183		36.1%		32/81	39.5%	0.594
Nodular	52/183		28.4%		24/81	29.6%	0.841
Diffuse	31/183		16.9%		20/81	24.7%	0.141
**CE (36- AND 60-h** **DIVIDE)**	**0–36 h**		**37–59 h**		**60-h**		***p*** **-Value (Chi-Sq)**
	**Patients, n (of Total)**	**Present (%)**	**Patients, n (of Total)**	**Present (%)**	**Patients, n (of Total)**	**Present (%)**	
No CE	22/60	36.7%	25/182	13.7%	1/22	4.5%	<0.001
Thin linear	24/60	40.0%	108/182	59.3%	17/22	77.3%	0.004
Thick linear	19/60	31.7%	70/182	38.5%	9/22	40.9%	0.594
Nodular	12/60	20%	57/182	31.3%	7/22	31.8%	0.231
Diffuse	7/60	11.7%	34/182	18.7%	10/22	45.5%	0.003

**Table 2:** The distribution of resection status before and after 48-h.

RESECTION STATUS	0–47-h		48-h		*p*-Value (Chi-Sq)
	Patients, n (of Total)	Present (%)	Patients, n (of Total)	Present (%)	
No CE tumour	66/218	30.3%	24/93	25.8%	0.426
No measurable tumour	117/218	53.7%	57/93	61.3%	0.215
Measurable tumour	35/218	16.1%	12/93	12.9%	0.477
No measurable tumour: Certain or uncertain of residual tumour					
Certain	90/117	76.9%	38/57	66.7%	0.150
Uncertain	27/117	23.1%	19/57	33.3%	

## Data Availability

The data presented in this study are available on request from the corresponding author. The data are not publicly available due to privacy restrictions.
